# Conceptualizing Callous-Unemotional Traits in Chinese Preschoolers: Factor Structure and Measurement Invariance

**DOI:** 10.3390/children10060925

**Published:** 2023-05-24

**Authors:** Gengli Zhang, Yantong Zhu

**Affiliations:** 1 Faculty of Educational Science, Anhui Normal University, Wuhu 241000, China; 2 School of Comprehensive Human Science, University of Tsukuba, Tsukuba 305-8577, Japan; zyt199431@gmail.com

**Keywords:** Chinese preschoolers, factor structure, inventory of callous-unemotional traits, measurement invariance, callous-unemotional traits

## Abstract

With the increasing use of the Inventory of Callous Unemotional Traits (ICU) to examine callous-unemotional traits, few studies have explicitly tested the most appropriate ICU factor structures and measurement invariance in Chinese children at preschool age. This study was conducted with a large community sample of 2055 Chinese preschoolers (53.6% male, M age = 62.23 months, SD = 9.91) to test the most appropriate model of ICU and the measurement invariance across parent gender, child gender, as well as age. The confirmatory factor model suggested that the two-factor model with 11 items (ICU-11) is the best-fitting model for a Chinese preschool sample, which includes a callousness and an unemotional factor. The results from measurement invariance revealed that the factor structures were invariant across child gender, as well as child age and parental gender. The finding suggested that the ICU-11 may be a useful tool for evaluating CU traits in Chinese preschoolers.

## 1. Introduction

Callous-unemotional (CU) traits reveal a unique subset of children at risk of severe conduct problems (CP), in addition to the risks associated with other early indicators of CP, such as oppositional-defiant behaviors [1]. The Diagnostic and Statistical Manual of Mental Disorders (DSM-5) has recently included CU traits as a specifier for conduct disorder (CD) (i.e., “with Limited Prosocial Emotions”) [2,3]. CU traits refer to having low empathy and limited prosociality [1,3]. Studies have emphasized that CU behaviors emerge as early as 2–3 years of age, and the foundations of CU behaviors, such as low empathy, develop early in childhood [4,5]. CU traits in early childhood are associated with severe behavioral problems later in life [6]. Significantly, CU traits have been found to be more treatable in early childhood than in adolescence [3,7]. Therefore, measuring the CU traits in early age and intervening earlier may minimize the probability of children developing more severe types of CP later [8].

The Inventory of Callous-Unemotional Traits (ICU), developed by Frick (2004), was developed to measure CU traits from preschoolers to old adults. The preschooler’s version compromises 24 items and 3 subscales: callous (e.g., does not seem to know “right” from “wrong”), uncaring (e.g., seems motivated to do his/her best in structured activities), and unemotional (e.g., does not show emotions) [9]. While significant research has been conducted on the child and youth versions of the ICU, as well as the CU traits being increasingly studied in younger children, the factor structure of ICU for early childhood at preschool age, especially in Eastern countries, is rare [8,10,11,12].

Previous research has utilized confirmatory factor analyses; however, no clear consensus has emerged [12]. The initial study offered a three-factor bifactor model in which all items were assigned to a general factor as well as three specific factors: callousness, uncaring, and unemotional [13]. Previous research applied this model to preschooler samples; however, the result indicated a poor model fit [8,10,11,12]. Ezpeleta, et al. [10] suggested a three-factor structure with correlated factors in preschoolers aged four to six. The contrast between items, which define empathic-prosocial (EP) versus CU behaviors, according to Willoughby, et al. [14], indicated theoretical and practical implications. Consequently, Willoughby, et al. [14] utilized confirmatory factor analysis to argue that a two-factor model distinguishing empathic-prosocial (EP) from callous-unemotional (CU) behaviors fitted the data in a Grade 1 community sample the best. Item response theory approaches were utilized by Hawes, et al. [7] to create a more psychometrically sound and efficient short form of the ICU, which consisted of two factors (i.e., callousness and uncaring) and consisted of 12 of the original 24 items. Other studies have replicated this finding in the US and Europe (i.e., Germany) on preschooler samples, through confirmation factor analysis and network analysis, and support the 12 items two factors model [8,11,12]. To reduce the impact of response sets by making informants take into account the direction of ratings across items, the original 24-item ICU had 12 positively and 12 negatively worded items [15]. The effect of positively and negatively worded items on distinct components is especially important in evaluating whether CU qualities are best considered a multidimensional or unidimensional construct [16].

Ignoring measurements would hamper test validity in terms of score comparability, cross-informant agreement, and incremental validity [17]. Currently, there are no existing studies that test the measurement invariance for parent-reported ICU across child gender, age, and parental gender (i.e., father and mother) among a Chinese preschool sample. Child age and gender are important factors to take into account because most child psychopathology displays large and relevant variations as a result of these variables [8]. ICU scores from a large representative sample of Greek children were higher in boys than in girls [18,19], according to a previous study that found that the factor structure of CU traits varied between boys and girls [20]. Girls display more severe internalizing difficulties, whilst boys display more severe CU traits and concomitant externalizing problems [21]. Additionally, it has been proposed [22] that the factor structure of CU traits may change as a child develops. Throughout childhood, emotional expression and control evolve and may affect the unemotional component [23,24,25]. The structure of the ICU may alter between mothers and fathers, as evidenced by research that suggests that moms may assess their child’s CU traits as more severe than fathers [8]. Early childhood CU traits are increasingly being explored [5,26]. However, few studies, particularly in Eastern cultures, have demonstrated the conceptualization of callous unemotional traits among preschoolers and the measurement invariance of ICU. This study aims to reveal the factor structure in a large community sample of Chinese preschoolers. In addition, our study aims to test the measurement invariance of ICU across preschoolers’ gender, age, and parental gender.

## 2. Materials and Methods

### 2.1. Sample and Procedure

According to the socioeconomic and child population density of the area, data were gathered from Wuhu City (seven districts, which included urban and rural areas) in Anhui Province, China. A total of 2055 preschoolers made up the sample (males made up 53.6%). Mothers of preschoolers completed 84.4% of the reports on the ICU. The preschoolers’ family’s annual income ranged from about ¥50,000 to over ¥300,000, with an estimated average income of between ¥100,000 and ¥150,000. Over the poor family income level, 93.1% of the families made more than 50,000 Yuan annually [27,28]. Table 1 provides descriptive data for the study’s background characteristics and all its variables. The study’s objectives were communicated to the principal of the school as well as the teachers of the students. Parents received web-based information and parental consent forms. Parents were all informed of the research goals, methods, and had the right to discontinue this study at any time. A total of 2055 parent-child pairs consented to participate in our study. An online survey from the “WenJuanXing” platform was used after collecting consent forms. All 2055 online questionnaires were sent to us, and all of the parents gave valid information that was used in subsequent data analysis. Although this study included a large sample, the research carried out is of a pilot study nature. The ethics committee at Anhui Normal University gave its approval for the project.

### 2.2. Measures

#### 2.2.1. Callous-Unemotional Traits

The 24-item parent-reported preschool-version ICU was used in this study to measure preschoolers’ CU traits [9]. It yielded scores on three dimensions: uncaring (e.g., seems motivated to do his/her best in structured activities), callousness (e.g., does not seem to know “right” from “wrong”), and unemotional (e.g., does not show emotions). Each item was evaluated on a four-point Likert scale ranging from 0 (not at all true) to 3 (absolutely true). Parents evaluated how well a statement described their child. The Cronbach’s α for the callousness, uncaring, unemotional subscales, and total score were 0.813, 0.649, 0.782, and 0.875, respectively.

#### 2.2.2. Demographic Information

Demographic information, such as children’s age, gender, and parental gender was reported by parents who filled out the online questionnaire. Parents indicated the children’s age, gender (1 = boy, 2 = girl), and their role (1 = mother, 2 = father) in the online questionnaire.

### 2.3. Statistical Analysis

First, the CFA was performed to explore the factor structure of CU traits in Chinese preschoolers. According to the ICU factor structure models reported in existing literature, eight different confirmatory factor analyses were used to see which model best fit our preschool sample: (Model 1) a single factor undifferentiated model; (Model 2) a 12-item two-factor model with the factors callousness and uncaring [7]; (Model 3) an 11-item two-factor model with the factors callousness and uncaring. Item 6 was deleted to fit the Chinese culture [29]; (Model 4) a 24-item, two-factor model with the factors callous-unemotional and empathic/prosocial [14]; (Model 5) a 22-item two-factor model with positively and negatively worded items [16]; (Model 6) a 24-item, three-factor model with the factors callousness, uncaring, and unemotional [10]; (Model 7) a 24-item, three-factor-higher-order hierarchical model with the factors general, callousness, uncaring, and unemotional [30]; and (Model 8) a 24-item, bifactor model with the factors general, callousness, uncaring, and unemotional [15].

Mean and variance-adjusted weighted least squares were used for rank ordinal data in accordance with earlier work [6,7,8,14]. To evaluate the model fit, the following metrics were used: the Tucker-Lewis Index (TLI), the Standardized Root Mean Square Residual (SRMR), the Comparative Fit Index (CFI), and the Root Mean Square Error of Approximation (RMSEA) (Hu & Bentler, 1999 [31]; Kline, 2015 [32]; MacCallum & Austin, 2000 [33]). Using the established benchmark values, the good model fit (CFI/TLI > 0.95, SRMR/RMSEA.08) and the acceptable model fit (CFI/TLI > 0.90, SRMR/RMSEA.10) were determined [34].

The measurement invariance for the best-fitting model across child gender, age, and parent gender is examined in the following phase. Chen [35] asserts that metric invariance may be created when compared to the configuration model, where CFI = 0.010, RMSEA = 0.015, and SRMR = 0.030, and scalar invariance can be attained when compared to the configuration model, where CFI = 0.010, RMSEA = 0.015, and SRMR = 0.010. The Statistical Package for the Social Sciences (SPSS), version 27 (Armonk, NY, USA), and Mplus 8.6 (Muthén & Muthén, Los Angeles, CA, USA) were used for all analyses.

## 3. Results

### 3.1. Descriptive Information

Table 1 presents the descriptive information for the main variables. The mean and standard deviation of age among baseline-year preschoolers was 57.4 ± 9.49 months. Of the total sample, 6.4% (n = 132) were age 3, 35.7% (n = 733) were age 4, 35.6% (n = 732) were age 5, 22.3% (n = 458) were age 6, and 1101 (53.6%) children were boys, while 954 (46.4%) were girls. Most questionnaires were reported by mothers (n = 1735, 84.4%), and others by fathers (n = 320, 15.6%).

### 3.2. CFA Models

Table 2 showed the model fit information of the eight models. Model 2 and Model 3 have acceptable model fit, as the other models demonstrated the worse fit. Compared to Model 2 (TLI = 0.977, CFI = 0.981, SRMR = 0.029, and RMSEA = 0.053), Model 3 showed a slightly better model fit (TLI = 0.981, CFI = 0.985, SRMR = 0.025, and RMSEA = 0.051). According to several studies which used Chinese-speaking samples [29,36,37], Item 6 (does not show emotions) may not be appropriate for evaluating CU traits in Chinese-speaking participants since it may be influenced by the Chinese cultural norm of hiding one’s emotions from others. After considering model fit and Chinese culture, we selected Model 3 as the best model. Table 3 shows the factor loadings for Model 3. The internal consistency of Model 3 was good for total scale (α = 0.82), callousness, and uncaring (α = 0.78, 0.74).

### 3.3. Measurement Invariance

Measurement invariance testing results of the ICU-11 by child gender, age, and parental gender are shown in Table 4. The configural models for child gender, age, and parental gender demonstrated excellent fit in the ICU-11 (child gender: CFI = 0.971, RMSEA = 0.042, SRMR = 0.028; child age: CFI = 0.963, RMSEA = 0.048, SRMR = 0.035; parental gender: CFI = 0.973, RMSEA = 0.041, SRMR = 0.028). There was no substantially worsened fit for either scale when equality constraints were applied to the factor loadings (metric model) or item intercepts (scalar model).

## 4. Discussion

The primary purpose of this study was to reveal the best-fitting factor structure of ICU, which is a widely used assessment tool for CU traits, among a large community preschooler sample in a Chinese cultural context. This study also examined the measurement invariance for ICU across child gender, age, and parental gender. Our study extended the previous study in two ways. First, this study’s findings validated a model with an 11-item, two-factor structure that was most appropriate for Chinese preschoolers. Second, we found that the ICU-11’s structure was consistent across child gender, age, and parental gender, indicating that the structure of CU traits does not differ between boys and girls, children of different ages (between 3 and 6 years old), or between parents of different genders.

Inconsistencies have been found in the literature on the definition of the construct of CU traits using the ICU in samples of different ages of children. However, studies using factor analysis on young children in their preschool years are rarely present. Previous research has shown that it is critical to specify a model before analyzing relationships. Similarly, models for samples containing older individuals cannot be presumed to be transferable to younger children without being evaluated [12]. Eight models extracted from previous studies were tested in this study through CFA, 11-item, and 12-item two-factor models with callousness and uncaring showed a good model fit [7,29], which is consistent with previous results utilizing Western preschool samples [8,12]. The three-factor model of the ICU (callousness, uncaring, and unemotional) [9] and the most widely used three-factor-bifactor model [15] both had poor model fits and were, therefore, not supported by our data. According to Cardinale and Marsh [21], methodological explanations (based on item pooling and wording) [38] can be used to explain why the unemotional sub-scale is weak. However, it may be inaccurate to categorize children with CU traits as “unemotional” given that this population struggles to manage their distress and has higher rates of negative affect [7,14,39]. Instead of being deficient in emotional expressiveness, these children may be displaying a reduced aptitude for prosocial emotion [6]. This raises the question of whether the term “Limited Prosocial Emotions”, as it appears in the DSM-5, ought to be utilized more frequently in place of CU, as it might be a better conceptualization of the construct during early childhood [8]. The 11-item and 12-item two-factor models with callousness and uncaring include similar items except for the 11-item model, which excludes item 6 of ICU, and thus showed a similar good model fit. The 11-item model had a better model fit and was more appropriate for the Chinese culture. One argument is that not showing emotion to others may be due to a lack of emotion or just a refusal to do so [29]. Parents might have perceived this as shy behavior rather than “lacking emotion” in the CU sense [7]. Another explanation is that children may be influenced by the Chinese cultural habit of keeping emotions hidden from others [29].

The structure of the ICU was also shown to be consistent across child gender, age, and parental gender in this study. Similar to previous research [8], the factor structure and strength of factor loadings of the ICU 11 were equivalent across boys and girls [29,40], allowing ICU-11 scores to be directly compared between preschool boys and girls. To guarantee the precise identification of at-risk children for intervention, it is crucial that assessments show gender equivalency [40]. Our results also revealed measurement invariance across age in Chinese preschoolers when using parent-reported ICU-11, which was consistent with a previous study [10]. This suggests that researchers and clinicians can use the ICU-11 to measure Chinese preschoolers’ CU traits across 3–6 and make direct comparisons of scores. Furthermore, we also found full measurement invariance across parental gender, which was consistent with a previous study [8]. This suggests that researchers and clinicians can utilize the ICU to compare CU traits between mothers and fathers, combining data from different types of informants.

Nonetheless, this study has some limitations. First, the sample of this study was recruited from a community sample. Therefore, we suggest that further studies could include clinical, forensic, or adjudicated samples. Secondly, this study only collected data from the parents. Psychopathological assessments usually had informant discrepancies which were found in a previous study when measuring CU traits [3]. We suggested future studies should examine the factor structures of teacher-reported ICUs and the measurement invariance between parent-reported and teacher-reported ICUs. Fourth, although most of the parents hold a secondary vocational or high school diploma and above, however, some of the parents with a low level of education may incorrectly assess the symptoms of emotional disorders in their children. Furthermore, some children may have symptoms of disorders belonging to other diagnostic categories, e.g., autism spectrum disorders, which could be mistakenly treated as symptoms of CU traits. Finally, this study used a cross-sectional study design to conceptualize the factor structures of ICU and measurement invariance. Future studies could extend a longitudinal measurement invariance to test the factor structures of ICU [29].

## 5. Conclusions

CU traits have been increasingly researched in preschoolers. It, therefore, warrants an exploration into the best model for ICU, which is the most widely used tool for assessing CU traits. This study expanded on prior research by investigating the best structure model of ICU for a Chinese preschooler sample. The findings showed that an 11-item two-factor model with callousness and unemotional factors is the best-fitting model, and it was invariant across child gender, age, and parental gender. These findings demonstrate that the ICU-11 may be a promising assessment tool that can be used for assessing CU traits in Chinese preschoolers.

## Figures and Tables

**Table 1:** Descriptive analysis for main variables.

Variables	Category	n (%) or Mean ± SD
Children age	3	132 (6.4)
	4	733 (35.7)
	5	732 (35.6)
	6	458 (22.3)
Children gender	Male	1101 (53.6)
	Female	954 (46.4)
Responds	Father	320 (15.6)
	Mother	1735 (84.4)
Parental age		34.87 ± 4.46
Father’s occupation	Farmers, nontechnical workers, and unemployment	79 (3.8)
	working with semi-technology and running a small business	558 (27.2)
	Worker in technology and semi-pro	530 (25.8)
	Professionals, officers, and midsize business proprietors	597 (29.1)
	high-level administrators and professionals	291 (14.2)
Mother’s occupation	Farmers, nontechnical workers, and unemployment	436 (21.2)
	working with semi-technology and running a small business	374 (18.2)
	Worker in technology and semi-pro	524 (25.5)
	Professionals, officers, and midsize business proprietors	609 (29.6)
	high-level administrators and professionals	112 (5.5)
Father’s education level	primary education or less	12 (0.6)
	lower middle school	259 (12.6)
	a secondary vocational or high school diploma	376 (18.3)
	degree from a technical college	480 (23.4)
	undergraduate degree	817 (39.8)
	Master’s or higher degree	111 (5.4)
Mother’s education level	primary education or less	23 (1.1)
	lower middle school	333 (16.2)
	a secondary vocational or high school diploma	349 (17.0)
	degree from a technical college	516 (25.1)
	undergraduate degree	744 (36.2)
	Master’s or higher degree	90 (4.4)
Family annual income	<¥50,000	142 (6.9)
	¥50,001–¥100,000	449 (21.8)
	¥100,001–¥150,000	613 (29.8)
	¥150,001–¥300,000	635 (30.9)
	>¥300,000	216 (10.5)

**Table 2:** Fit indices for eight models.

Model	χ^2^	DF	CFI	TLI	RMSEA	SRMA
One-factor (undifferentiated)	5539.801	252	0.840	0.824	0.101	0.083
Two-factor (callousness, uncaring; 12 items)	355.736	53	0.981	0.977	0.053	0.029
Two-factor (callousness, uncaring; 11 items)	273.420	43	0.985	0.981	0.051	0.025
Two-factor (callous-unemotional, empathic/prosocial; 24 items)	3931.131	251	0.888	0.877	0.084	0.065
Two-factor (positive and negative item)	8042.529	208	0.743	0.715	0.135	0.109
Three-factor (callousness, uncaring, and unemotional; 24 items)	4402.682	249	0.874	0.860	0.090	0.070
Three-factor-higher-order hierarchical model (General, callousness, uncaring, and unemotional; 24 items)	2584.048	249	0.787	0.764	0.068	0.065
Bifactor (General, callousness, uncaring, and unemotional; 24 items)	6267.722	232	0.817	0.782	0.113	0.087

**Table 3:** The factor structure of ICU-11.

	Callousness	Uncaring
Item 3	0.645	
Item 9	0.599	
Item 11	0.699	
Item 12	0.750	
Item 18	0.800	
Item 21	0.736	
Item 5		0.604
Item 8		0.721
Item 16		0.659
Item 17		0.815
Item 24		0.692

**Table 4:** Measurement invariance for ICU-11 across child gender, age, and parental gender.

Model	χ^2^	DF	CFI	ΔCFI	RMSEA	ΔRMSEA	SRMA	ΔSRMR
Child gender								
Configural invariance	244.756	86	0.971		0.042		0.028	
Metric invariance	258.551	95	0.970	−0.001	0.041	−0.001	0.032	0.004
Scalar invariance	268.600	104	0.970	0.000	0.039	−0.002	0.032	0.000
Child age								
Configural invariance	375.316	172	0.963		0.048		0.035	
Metric invariance	401.923	199	0.963	0.000	0.045	−0.003	0.041	0.006
Scalar invariance	431.762	226	0.963	0.000	0.042	−0.003	0.042	0.001
Parental gender								
Configural invariance	232.841	86	0.973		0.041		0.028	
Metric invariance	254.062	95	0.971	−0.002	0.040	−0.001	0.033	0.005
Scalar invariance	259.830	104	0.971	0.000	0.038	−0.002	0.034	0.001

## Data Availability

Not applicable.
